# Preparation, Characterization, and In Vitro Digestion Behavior of Alginate–Chitosan Microspheres Loaded with *Ziziphus jujuba* Pulp

**DOI:** 10.3390/foods15030594

**Published:** 2026-02-06

**Authors:** Dan Zhao, Nannan Chen, Beizhi Zhang, Fuzhi Xie, Qing Zhang, Bei Fan, Xiaona Liu, Ziguo Rong, Min Ju, Mengmeng Yu, Yongchang Dai, Fengzhong Wang, Liang Zhang

**Affiliations:** 1 National Center of Technology Innovation for Comprehensive Utilization of Saline-Alkali Land, Dongying 257000, China; bszhaodan@163.com (D.Z.); chennan0114@gmail.com (N.C.); 2 Institute of Food Science and Technology, Chinese Academy of Agricultural Sciences, Beijing 100193, China; zhangbeizhi777@163.com (B.Z.); xiefuzhi2002@163.com (F.X.); 13345033761@163.com (Q.Z.); fanbei517@163.com (B.F.); 3 College of Food Science and Engineering, Qingdao Agricultural University, Qingdao 266000, China; 4 College of Food Science and Technology, Huazhong Agricultural University, Wuhan 430070, China; 5 Service Center for Comprehensive Utilization of Saline-Alkali Land in Agricultural High-Tech Industrial Demonstration Zone of the Yellow River Delta, Dongying 257000, China; hsjngqkjcxj@shandong.cn (X.L.); rongzg123456@163.com (Z.R.); jm95311@163.com (M.J.); 6 Shandong Huibangbohai Agricultural Development Co., Ltd., Dongying 257000, China; yumengmeng214@163.com; 7 Dongying Guangyuan Biotechnology Co., Ltd., Dongying 257000, China; li@gyswvip.com; 8 Institute of Food and Nutrition Development, Ministry of Agricultura and Rural Affairs, Beijing 100081, China

**Keywords:** *Ziziphus jujuba*, alginate–chitosan, microspheres, pH-responsive release, in vitro digestion, amorphous solid dispersion, antibacterial activity

## Abstract

In this study, sodium alginate–chitosan composite microspheres (S-C Ms) were prepared by ionic gelation to encapsulate *Ziziphus jujuba* pulp from wild jujube pulp. The effects of sodium alginate (SA) concentration, chitosan (CS) concentration, and core-to-wall ratio on encapsulation efficiency (EE%) and loading capacity (LC%) were systematically investigated. The results showed that both EE% and LC% were maximized when the SA concentration was 2.0% (*w*/*v*) and the CS concentration was 1.5% (*w*/*v*). The FTIR and XRD analyses confirmed the successful encapsulation of a phenolic-rich extract from *Z. jujuba* pulp (PRE) and its transformation into an amorphous state, while the SEM observations revealed that the composite microspheres possessed a well-defined morphology and a dense internal structure. Particle size analysis further indicated a narrow and uniform size distribution. Thermogravimetric analysis (TGA) and in vitro simulated digestion demonstrated that S-C Ms exhibited a pH-responsive release profile, characterized by slow, limited release in the gastric phase and markedly enhanced release in the intestinal phase. The release mechanism in simulated gastric fluid was dominated by Fickian diffusion, whereas it shifted to an erosion-controlled process in simulated intestinal fluid. Consistently, the swelling ratio of the microspheres was low at pH 1.2 but increased sharply at pH 7.0, reflecting a “gastric protection–intestinal release” behavior. Antibacterial assays showed that P-loaded microspheres exerted significant inhibitory effects against *Staphylococcus aureus* and other test strains, with the antibacterial activity possibly associated with the controlled release during the in vitro digestion of compounds with antimicrobial potential, such as phenolic compounds. Overall, SA-CS composite microspheres exhibited favorable encapsulation performance, structural stability, and controlled-release potential, making them a promising delivery and protection system for *Ziziphus jujube* pulp bioactive compounds.

## 1. Introduction

In today’s society, the sustainable development of biomass energy and the high-value utilization of agricultural waste have become urgent global issues. The United Nations Environment Programme (UNEP) released the Food Waste Index 2024 report, which pointed out that a large amount of food is still discarded along the production and consumption chain. In 2022 alone, up to 1.05 billion tons of food were ultimately wasted worldwide, accounting for about one-fifth of the total amount of food available to consumers [1].

Jujube is a typical example within the broader context of high-value utilization of global agricultural waste. *Ziziphus jujuba* Mill., a woody plant of the family Rhamnaceae, is an important traditional medicinal and edible species that is mainly distributed in the Taihang Mountain area of China, particularly in Xingtai, Hebei Province. Studies have shown that the kernel of *Z. jujuba* has sedative, hypnotic, and antidepressant effects; it is often used to treat insomnia and improve sleep quality [2]; thus, most kernels are processed in traditional Chinese medicine [3]. By contrast, a large amount of jujube pulp is generated as processing waste, and is usually discarded or used in low-efficiency applications. Specifically, in the industrial processing of *Ziziphus jujube* for kernel extraction (the primary step for its medicinal utilization), fresh fruits are first subjected to washing and mechanical comminution, followed by physical separation (e.g., vibratory sieving or density-based centrifugation), to isolate the rigid kernels. The resultant residual fraction—comprising the fruit’s pericarp and mesocarp, collectively termed *Ziziphus jujube* pulp—remains unexploited post-kernel recovery, owing to the current lack of scalable high-value utilization technologies [4].

However, compared with the kernel, the pulp is also rich in nutrients and contains nucleotides, amino acids, polysaccharides, vitamins, and a variety of bioactive compounds such as phenols, flavonoids, saponins, and terpenoids [5,6,7], indicating good application potential. Previous studies have shown that jujube pulp is the main source of aromatic glycoside polyphenols in jujube [8]. Nevertheless, the bioactive compounds in jujube pulp exhibit poor stability, are easily oxidized, and show unsatisfactory digestive performance in the human gastrointestinal tract [9]. Considering both the benefits of jujube pulp bioactive compounds and these challenges, it is essential to develop an efficient encapsulation system for them.

Microsphere technology is a widely recognized encapsulation strategy. Sodium alginate (SA) is a natural, biodegradable, and biocompatible polysaccharide. Owing to its excellent gel-forming ability, SA has been widely used as a wall material for microsphere encapsulation [10,11]. However, microspheres formed solely by calcium ion-crosslinked sodium alginate have obvious limitations [12]. First, the internal voids after crosslinking are relatively large, which easily leads to the leakage of encapsulated material [13]. Second, sodium alginate displays poor stability in the strongly acidic gastric environment and is prone to degradation, resulting in the premature release of encapsulated compounds and reduced bioavailability [14,15,16]. To address the limitations of single alginate microspheres, composite microsphere systems based on electrostatic interactions were investigated. Chitosan (CS) is a natural polymer bearing positive charges. It can combine with negatively charged sodium alginate to form sodium alginate–chitosan composite microspheres via ionotropic gelation. This composite structure can form a more stable capsule wall and significantly reduce leakage [17,18,19]. Moreover, due to the acid-protective effect of chitosan, the capsules are less susceptible to degradation in the gastric environment [20], which facilitates the targeted release of active ingredients in the intestine and improves their bioavailability [21]. While the bioactive compounds of *Z. jujuba* pulp exhibit diverse biological functions, including significant antioxidant capacity, their antimicrobial potential is particularly critical for enhancing food safety and extending shelf-life. Considering the persistent challenges posed by foodborne pathogens such as *Staphylococcus aureus* and *Escherichia coli*, developing a delivery system that can ensure the controlled release and sustained efficacy of these antimicrobial agents is highly desirable. Therefore, in addition to structural characterization and digestion behavior, this study specifically evaluates the antibacterial activity of the developed microspheres to demonstrate their practical potential as a functional delivery platform for food preservation.

To mitigate the underutilization of jujube pulp waste and harness its inherent bioactive properties, this study aims to fabricate sodium alginate–chitosan composite microspheres via ionic gelation for the encapsulation of jujube pulp bioactives. The study hypothesizes that this pH-responsive carrier system will enhance the stability and site-specific delivery of these bioactives; the novelty of this work resides in the integration of underutilized jujube pulp waste into a functional encapsulation platform tailored for controlled release and antibacterial applications.

## 2. Materials and Methods

### 2.1. Raw Materials and Reagents

A phenolic-rich extract (PRE) from *Z. jujuba* pulp was prepared in laboratory. Briefly, the pulp was prepared in our laboratory using a Deep Eutectic Solvent (DES) extraction method, as previously described by Zhao et al. [4]. Sodium alginate (SA) (analytical-grade, purity > 99.7%) and chitosan (CS) (analytical-grade, purity > 99%) were purchased from Shanghai Aladdin Biochemical Technology Co., Ltd. (Shanghai, China). Calcium chloride (CaCl_2_), anhydrous ethanol, and other chemical reagents were obtained from Sinopharm Chemical Reagent Co., Ltd. (Shanghai, China). Dilute acid and gallic acid standards (≥99% were purchased from Hebei Bailingwei Ultra-fine Materials Co., Ltd. (Langfang, China). Folin–Ciocalteu reagent and phosphate-buffered solution (PBS) (pH 1.2, 5.0, 7.0, 7.4) were acquired from Beijing Solarbio Science & Technology Co., Ltd. (Beijing, China). Deionized water and ultrapure water were prepared using a HEAL DORCE NW ultrapure water system (Hong Kong Likang Biomedical Technology Holdings Group Co., Ltd. Hong Kong, China). Potassium bromide (spectral pure purity > 99.7%) was obtained from Shanghai Aladdin Biochemical Technology Co., Ltd. For microbiological assays, *Staphylococcus aureus* (Gram+) (ATCC 29213) and *Escherichia coli* (Gram−) (ATCC 25922) were used. Mueller–Hinton (MH) agar medium was purchased from Shanghai Aladdin. Simulated gastric fluid (SGF) (pH 1.2) (USP, sterile, pepsin concentration of 3.2 g/L) and simulated intestinal fluid (SIF) (pH 8.8) (sterile, trypsin concentration of 0.464 g/L) were purchased from Shanghai Yuanye Bio-Technology Co., Ltd. (Shanghai, China).

### 2.2. Experimental Methods

#### 2.2.1. Preparation of Polyphenol Microspheres Using Ion Gel Method

Polyphenol-loaded microspheres were prepared using the ionotropic gelation method, with slight modifications in the previously reported alginate–chitosan encapsulation method [20]. The active substances of the phenolic-rich extract from *Z. jujuba* pulp (PRE) to be embedded were fully dissolved in 2% (*w*/*v*) SA aqueous solution and mixed evenly to form a core solution loaded with PRE. Subsequently, the pH of the 1.5% (*w*/*v*) chitosan solution was adjusted to 5.0 with dilute acid, and then it was mixed with 5% (*w*/*v*) calcium chloride (CaCl_2_) aqueous solution as an external curing medium. In the process of microsphere formation and curing, the curing solution was placed in a beaker and stirred continuously at 400 r/min at room temperature with magnetic force. Then, the core solution was added dropwise at a constant speed using a syringe into the solidifying liquid. Afterwards, a compound SA and CS cement layer was formed via electrostatic interactions and Ca squared ^+^ ionic crosslinking with SA carboxyl, prompting rapid solidification. The solution drops continued to be added after the completion of slow mixing for 30 min to ensure that the crosslinking was complete; then, the liquid was allowed to stand for 20 min for microsphere settlement, and the mother liquor was separated with filtration. After separation, the microspheres were washed three times with deionized water to remove surface impurities. In each cycle, separation was achieved by centrifugation at 3000 rpm for 5 min at 4 °C. The entire volume of the filtrate and washed solution was collected, and the total volume was accurately recorded for subsequent analysis. Finally, dry *Z. jujuba* bioactive compound microspheres (sodium alginate–chitosan microspheres, S-C Ms) were obtainedat a constant weight by freeze drying and vacuum drying treatment at −80 °C; among these microspheres, there were microspheres with drug compounds (the PRE-loaded sodium alginate–chitosan microspheres, S-C Ms-1) and without drug compounds (unloaded sodium alginate–chitosan microspheres, S-C Ms-0).

#### 2.2.2. Determination of Embedding Rate and Drug Loading of Microspheres

The embedding rate and drug loading of microspheres are calculated by determining the free polyphenols. Specifically, 10 mg of microspheres are weighed and added to distilled water or a buffer solution (pH 7.0) for ultrasonic crushing to release the polyphenols from the core material. After the samples were centrifuged at 8000 rpm/min for 10 min, the supernatant was taken, and the concentration of polyphenols was determined using the Folin–Ciocalteu method [22]. Noteworthily, the Folin–Ciocalteu reagent is sensitive to various reducing agents other than phenolic compounds, such as certain sugars and organic acids that may be present in *Z. jujuba* pulp. Therefore, the results obtained from this analysis are interpreted as the total reducing capacity and serve as a general indicator of the bioactive compounds within the phenolic-rich extract (PRE). The encapsulation rate (EE %) and loading capacity (LC %) were calculated. The standard curve was y = 1.8527x + 0.0626, R^2^ = 0.999 [23].

(1) $$EE\left(\%\right)=\frac{Amount\;of\;bioactive\;compounds\;in\;the\;microspheres}{Amount\;of\;bioactive\;compounds\;initially\;added}\times 100\%$$

(2) $$LC\left(\%\right)=\frac{Amount\;of\;bioactive\;compounds\;in\;microspheres}{Total\;mass\;of\;microspheres}\times 100\%$$

#### 2.2.3. Investigation of Process Parameters

To explore the effect of SA and CS concentrations on the properties of microspheres and screen for suitable preparation conditions, in this study, the ionic gelation was used to prepare microspheres, and the single-factor experiments were carried out for systematic comparison [24]. The selection of concentrations was mainly evaluated based on the EE and the formation status (morphology and dispersibility) of the microspheres. Specifically, SA solutions with concentrations of 0.5%, 1.0%, 2.0%, 3.0%, and 5.0% (*w*/*v*) and CS solutions with concentrations of 0.5%, 1.0%, 1.5%, 2.0%, and 3.0% (*w*/*v*) were prepared. The accurately weighed SA powder was slowly added to deionized water, magnetically stirred at room temperature until completely dissolved to form a uniform transparent solution, and then left to stand to remove bubbles. SA-CS microspheres were then prepared as described in Section 2.2.1

#### 2.2.4. Characterization Using Fourier-Transform Infrared Spectroscopy

Chemical structure and embedding effect were characterized with Fourier-Transform Infrared Spectrometer (FTIR) (TENSOR 27, Bruker, Ettlingen, Germany) analysis. The S-C Ms were freeze-dried, and the water was completely removed. A 2 mg quantity of the dried sample and 200 mg of dried KBr were mixed in an agate mortar using the tabbing method, ground evenly, pressed into a transparent sheet by a tabbing machine under a pressure of about 10 MPa, and analyzed in the wavenumber range of 4000–400 cm^−1^. The number of scans was 32–64, with a resolution of 4 cm^−1^, contrasting with S-C Ms-0 and S-C Ms-1 [25].

#### 2.2.5. Thermal Stability

Thermogravimetric analysis (TGA) (TG/DTA6300, Seiko Instruments Inc., Chiba, Japan) was performed to evaluate the thermal stability of the samples. Approximately 5.0 to 10.0 mg of the dried microsphere samples was analyzed, using the SA/CS physical mixture (unreacted wall material) and S-C Ms-0 as the blank control, from room temperature to 600 °C at a heating rate of 10 °C/min under a nitrogen atmosphere. The weight loss percentage, decomposition temperature, and residual mass were calculated from the TG curves to analyze the thermal behavior of the microspheres [26].

#### 2.2.6. Morphology of Microspheres

The morphological characterization of S-C microspheres was performed to investigate their surface structure and embedding effect. The microspheres were quickly frozen (−80 °C) and vacuum freeze-dried. Then, an appropriate amount of the completely dried microsphere sample was taken, and a conductive double-side tape was used to firmly attach it to the scanning electron microscope (SEM) (SU8010, Hitachi, Tokyo, Japan) sample table. Afterwards, a uniform layer of gold film was sprayed on the surface of the sample with an ion sputtering instrument under an argon atmosphere. The prepared samples were placed in the sample chamber of the SEM, and the morphology was observed under the high-vacuum mode at an accelerating voltage of 5 kV. The SEM morphology of SA single-wall material (sodium alginate microsphere, SA Ms) was used as a control [27].

#### 2.2.7. Particle Size Analysis Using Laser Diffraction

The particle size distribution of the microspheres was determined using a Mastersizer 3000 laser particle size analyzer (Malvern Panalytical Ltd., Malvern, UK) [26]. During the measurement, ultrapure water was used as the dispersion medium. After the background signal was stabilized, the sample suspension dispersed by ultrasound was slowly injected into the sample circulation cell until the shading rate was in the optimal measurement range (10–20%). Each sample was measured 3 times in parallel at 25 °C. The particle size was expressed as D10, D50, and D90, representing the diameters at 10%, 50%, and 90% of the cumulative volume distribution, respectively. To characterize the polydispersity of the microspheres, the Span value was calculated using the following equation:

(3) $$Span=\frac{\left(D90-D50\right)}{D50}$$

#### 2.2.8. X-Ray Diffraction Analysis

The crystal structure and relative crystallinity of the samples were analyzed using an X-ray powder diffractometer (XRD) (D8 Advance, Bruker, Ettlingen, Germany) [28]. The specific steps are as follows: the freeze-dried sample was placed on the sample table and gently flattened to form a uniform plane. Determinations were performed using a D8 Advance X-ray diffractometer equipped with a Cu-Kα radiation source (wavelength λ = 0.154 nm). The test conditions were as follows: tube voltage = 40 kV; tube current = 40 mA; scanning range (2θ) = 5° to 50°; scanning step size = 0.02°; and scanning speed = 4°/min.

#### 2.2.9. Determination of Swelling Behavior

The swelling behavior of the microspheres at different pH was investigated using the gravimetric method [29]. The procedure was as follows: The freeze-dried microspheres of a certain mass (W_0_, about 10.0 mg) were precisely weighed and loaded into the pre-drying bag of known weight. The drying bags were completely immersed in beakers containing 50 mL phosphate-buffered solution (PBS) at pH 1.2, 5.0, and 7.0. All beakers were placed in a 37 °C thermostatic water bath shaker and shaken at 100 rpm/min. At the set time points (1, 2, 3, 4, 5, 6, 7, 8, 9, and 10 h), the drying bag was removed from the buffer, the excess liquid on its surface was carefully blotted with filter paper, and its total weight (Wt) was immediately weighed accurately with an analytical balance. Each weighing operation was completed within 30 s to minimize the evaporative loss of water. Subsequently, the sample was placed back into the original buffer to continue swelling. Each sample was determined 3 times in parallel, and the swelling kinetic curve, i.e., swelling rate against time, was plotted. The swelling ratio (SR) was calculated using the following formula:

(4) SRggWt−W0−WbW0

where W0 denotes the initial mass (g) of the dried microspheres; Wt denotes the total mass (g) of the swelling microspheres and the drying bag at time t; Wb denotes the mass of the blank wet-drying bag (g) [30]. To strictly evaluate the release mechanism and structural relaxation, the swelling data were further fitted to the Korsmeyer–Peppas, Higuchi, and Hixson–Crowell kinetic models to determine the diffusion exponent (*n*) and rate constants.

#### 2.2.10. In Vitro Simulated Digestion

The gastrointestinal stability and bioactive compounds release profiles of S-C Ms were investigated using a static in vitro digestion model, conducted in accordance with the United States Pharmacopeia (USP) guidelines [31]. For the gastric phase, 50 mg of microspheres were immersed in 10 mL of simulated gastric fluid (SGF, pH 1.2), which contained 3.2 g/L pepsin. The mixture was incubated at 37 °C with continuous agitation at 100 rpm for 4 h. Following gastric digestion, the mixture was centrifuged (5000 rpm, 10 min) to separate the released PRE. The resulting supernatant was collected for subsequent analysis, while the remaining precipitate was re-suspended in 10 mL of simulated intestinal fluid (SIF, pH 8.0). The SIF contained 0.464 g/L pancreatin and phosphate salts. The intestinal digestion proceeded under identical temperature and shaking conditions for an additional 4 h. Upon completion, the intestinal mixture was centrifuged, and the supernatant was harvested. The bioactive compounds concentration in the collected supernatants from both stages was quantified using the Folin–Ciocalteu method to determine the cumulative release rate [32,33,34].

(5) $$Bioactive\;compounds\;release\;rate\left(\%\right)=\frac{Amount\;of\;bioactive\;compounds\;released}{Amount\;of\;bioactive\;compounds\;loaded}\times 100\%$$

#### 2.2.11. Evaluation of Antimicrobial Activity

The agar diffusion method was used to evaluate the antibacterial effect of the microspheres. Following activation, the concentrations of *Staphylococcus aureus* (Gram+) and *Escherichia coli* (Gram−) suspensions were standardized to 0.5 McFarland turbidity units and subsequently diluted to a final concentration of 1 × 10^6^ CFU/mL. Separately, the microspheres were dispersed in sterile PBS (pH 7.4) to prepare the microsphere suspension. The S-C Ms-1 microspheres and sterile PBS were set as the blank control group. Then, the bacterial suspension was coated on the Mueller–Hinton agar plate, placed in a sterile Oxford cup, and 100 μL of sample was added. After incubation at 37 °C for 24 h, the diameter of inhibition zone was measured.

All experiments were performed in three replicates, and the results are expressed as mean values ± SD. Data analysis was performed using SPSS v26.0 and GraphPad Prism 10.

## 3. Results and Discussion

### 3.1. Determination of SA Concentration

**Figure 1:**
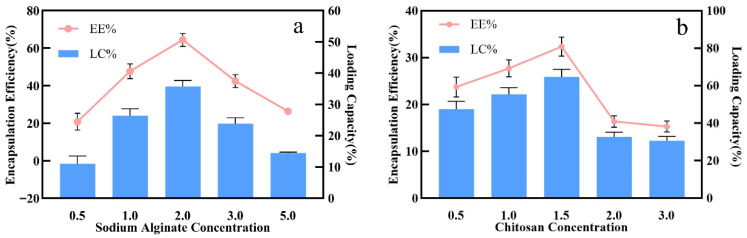
The effect of different concentrations of SA (**a**) and CS (**b**) on the embedding efficiency of microspheres.

The SA concentration has a significant effect on the embedding rate and drug loading and the formation status (size and morphology) of the microspheres (Figure 1a) [35]. The embedding rate and drug loading of microspheres change with the change in SA concentration, and both show a trend of an initial increase and then a decrease. Simultaneously, the viscosity-dependent droplet formation significantly influenced the particle size uniformity. When the concentration of SA was 2.0% (*w*/*v*), the optimal SA concentration was obtained, and the embedding rate and drug loading reached the maximum at the same time. At this time, DL% and EE% both reached the peak, which were 66.32% and 36.79%, respectively. Moreover, it was found that a too high or too low concentration of SA was not conducive to embedding. When the concentration of SA was low, such as 0.5% (*w*/*v*) and 1.0% (*w*/*v*), the embedding rate and drug loading were correspondingly low, which was mainly because, at a low SA concentration, the viscosity of the solution was insufficient, which made it difficult to form regular spherical droplets [36]. The resulting particles were fragile with poor sphericity. In the process of ion curing, the formed gel network structure was loose, and the pore size was large. This loose structure is not enough to effectively block the diffusion of internal PRE to the external media, resulting in a large amount of core material leakage during the curing and washing steps and, thus, low levels of EE% and LC% [37]. With the increase in SA concentration, the solution viscosity was appropriate, and the formed gel network became denser and stronger due to the increase in crosslinking sites (-COO^−^). At 2.0% (*w*/*v*), the microspheres exhibited the best sphericity and size uniformity, which could more effectively encapsulate PRE in the interior of the microspheres, thereby significantly improving the embedding effect. However, when the SA concentration was too high, such as 3.0% and 5.0% (*w*/*v*), the embedding efficiency and drug loading decreased. When the SA concentration was further increased from 2.0% to 5.0%, both EE% and LC% decreased sharply. When the SA concentration was 5.0%, EE% and LC% decreased to 26.25% and 14.33%, respectively. This was attributed to a sharp increase in the viscosity of the core solution caused by the excessively high SA concentration. Alternatively, this deterioration was attributed to the excessive viscosity, which hindered the formation of uniform droplets. The high viscosity prevents the solution from being syringed outuniformly and smoothly, affecting the formation of the microspheres; more importantly, the viscous gel system seriously hinders the diffusion and mass transfer of external curing agent Ca^2+^ to the inside of the droplet. This results in the rapid surface curing of the microspheres without sufficient internal crosslinking, resulting in the formation of an uneven “core–shell” structure. Therefore, considering the encapsulation efficiency, drug loading, and particle size uniformity (which is critical for consistent release kinetics), 2.0% *(w*/*v)* was selected as the optimal SA concentration, which leads to the internal PRE being easily leaked during subsequent processing. Therefore, the experimental results showed that the optimal SA concentration was 2.0% (*w*/*v*), at which the highest embedding efficiency and drug loading could be obtained [38].

### 3.2. Determination of CS Concentration

CS was used as an outer coating material to enhance the stability of the microspheres and further reduce the leakage of the core substance by electrostatic interactions with the carboxyl group on the surface of sodium alginate to form a layer of polyelectrolyte complex film [39,40,41]. As shown in Figure 1b, the concentration of CS also has a significant effect on the embedding performance of the microspheres. As the concentration of CS in the curing solution increased from 0.5% (*w*/*v*) to 1.5% (*w*/*v*), the EE% of the microspheres increased from about 23.88% to the peak of about 32.05%, and the LC% also increased from about 47.76% to the peak of about 64.11%. This is because, at lower concentrations, the number of CS molecules is insufficient to form a complete and dense coating on the surface of the SA microspheres. With the increase in CS concentration, the electrostatic adsorption between the positively charged amino group (-NH_3_^+^) and the negatively charged carboxyl group (-COO^−^) on the surface of SA is enhanced, forming a more effective physical barrier, promoting the construction of a more compact and stable wall network, blocking the pores of the gel network, thereby improving the interception ability of the microspheres to PRE [39,40,42].

Moreover, the EE% and LC% were increased. However, when the CS concentration exceeded 1.5%, EE% and LC% decreased significantly. When the CS concentration increased to 2.0% and 3.0%, EE% decreased sharply from 32.05% to about 15.97% at 2.0%, and LC% also decreased correspondingly. The mechanism may involve two factors: On the one hand, excessive CS introduces too much positive charge, which destroys the charge balance between the two phase polymers, leads to enhanced electrostatic repulsion, interferes with the formation of uniform and stable condensates, and even causes flocculation, leading to the aggregation of microspheres or the formation of too thick and uneven coating on the surface of microspheres. This structural defect may reduce the overall stability of microspheres. In the washing process, the coating falls off and leads to the loss of contents, thereby reducing the encapsulation efficiency. On the other hand, the viscosity of the system increased significantly due to the high concentration of CS, which affected the dispersion and morphological integrity of the microspheres in the curing process, thereby weakening the functional performance of the final product [39,42]. The results of this experiment show that the optimal composite condensation structure can be formed between SA and CS at a CS concentration of 1.5%, thus achieving the optimal balance between embedding efficiency and drug loading capacity.

### 3.3. Laser Particle Size Analysis

**Figure 2:**
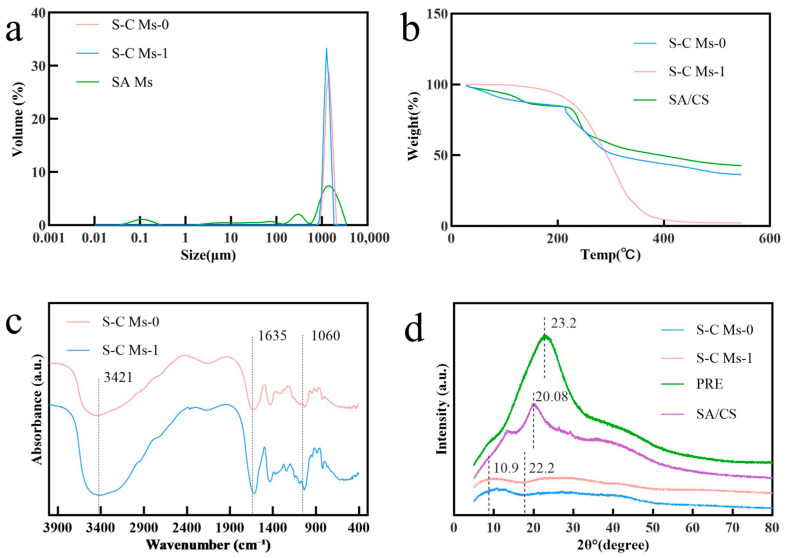
The physicochemical characterization of microspheres prepared with different wall materials: (**a**) the particle size distribution of the microspheres; (**b**) the differential thermogravimetric (DTG) analysis curves of the microspheres, indicating their thermal stability; (**c**) the Fourier-transform infrared (FTIR) spectra of the microspheres, showing their chemical functional groups; and (**d**) the X-ray diffraction (XRD) patterns of the microspheres, used to determine their crystalline nature.

The particle size and size distribution of microspheres are key quality attributes for their use as delivery vehicles, because they directly influence mobility, release kinetics, and suitability for specific applications [43,44]. The results in Figure 2a show that the prepared S-C microsphere samples exhibited a unimodal size distribution, indicating that the particle population was relatively narrow and homogeneous [45]. Specifically, the Span values were found to be in the range of 0.4 to 0.5, quantitatively confirming the uniform size distribution. The S-C Ms prepared with SA-CS composite wall materials displayed markedly better particle size uniformity and physical stability than those prepared with single SA walls. In addition, loading jujube pulp bioactive compounds into the core did not markedly shift the particle size distribution, indicating that the embedding process was highly stable and reproducible. Specifically, the volume fraction of the main peak region for polyphenol-loaded S-C Ms-1 was not significantly different from that of the unloaded S-C Ms-0, suggesting that the incorporation of the core material had no significant effect on the size distribution of the composite-wall microspheres. This further confirms that the ionotropic gelation process can generate composite microspheres with regular structures and robust performance, providing a favorable morphological basis for the effective encapsulation of active ingredients [44,45].

In contrast, SA Ms exhibited a typical multimodal distribution, with peaks around 0.1 μm, 100 μm, and 1000 μm, and the volume fraction at 1000 μm was much lower than that of the S-C Ms series. These results suggest that microspheres formed from single SA walls possess poorer size uniformity and insufficient control over droplet formation during the complex gelation process [41]. Upon the addition of CS, the resulting composite microspheres showed a sharp unimodal distribution with particle sizes concentrated around 1500 μm (Table 1). This behavior can be attributed to the dense polyelectrolyte complex film produced by electrostatic interactions between CS and SA, which markedly improves the structural integrity and mechanical strength of the microspheres, whereas single SA walls lack such strong interactions [46]. Overall, the SA-CS complexation system enables the uniform control of microsphere particle size while stably carrying active ingredients, thereby providing a reliable material platform and process basis for the microsphere-mediated delivery of functional factors.

### 3.4. Thermal Stability Analysis

Thermogravimetric analysis (TGA) was used to evaluate how the embedding of PRE within SA/CS microspheres affected their thermal stability. The results (Figure 2b) showed that the thermal behavior of sodium alginate–chitosan (SA/CS)-based microspheres and their physical mixtures differed significantly. Specific decomposition parameters, including weight loss stages and peak degradation temperatures, were analyzed. The physical mixture of S-C Ms-0 and SA/CS exhibited a moderate weight loss from 200 to 550 °C, with a carbon residue of approximately 35–45% at 550 °C. In contrast, microspheres loaded with PRE (S-C Ms-1) showed pronounced weight loss in the main pyrolysis region (an onset temperature of approximately 220–300 °C), and the residual mass at 400 °C was close to 51%. In the range of 150–200 °C, all samples showed slight weight loss due to the removal of adsorbed water and volatile substances, which is consistent with the typical thermal behavior of polysaccharide-based materials. These results indicate that the thermal stability profile of the microspheres changed significantly after drug loading, with the maximum decomposition temperature (T_max_) shifting from 232 °C (physical mixture) to 440 °C (S-C Ms-1) [47].

This difference in thermal behavior should be interpreted considering the functional goals of the encapsulation system. For natural active-ingredient delivery systems, the primary objective is to enhance the retention, bioavailability, and targeted release of the active ingredient rather than to merely maximize the thermal stability of the carrier. Non-encapsulated jujube bioactive compounds exhibited poor stability and low bioavailability, with an activity retention rate of only about 20% after storage at room temperature for 1 month and an oral bioavailability of less than 10%. Although the apparent high-temperature thermal stability of S-C Ms-1 decreased, its SA/CS polyelectrolyte composite structure improved the retention of bioactive compounds to 65.3% and achieved 67% cumulative release in simulated intestinal fluid, corresponding to an approximately 4.2-fold increase in bioavailability. This reflects a deliberate strategy in which a moderate compromise of carrier thermal stability is accepted in exchange for enhanced functionality, in line with the “function-first” design principle for active delivery systems [48,49].

The altered thermal response of S-C Ms-1 is attributed to molecular interactions between the bioactive compounds and the carrier. The phenolic hydroxyl and carboxyl groups of jujube pulp bioactive compounds can form hydrogen bonds and electrostatic complexes with the amino groups of chitosan and the hydroxyl/carboxyl groups of sodium alginate. These interactions disrupt the regularity of the original crosslinked network and reduce the activation energy required for chain scission, disrupting the regularity of the original crosslinked network and reducing the energy required for chain scission during pyrolysis, which manifests as intensified mass loss and a lower decomposition temperature in the main degradation stage [50,51]. Nevertheless, the microsphere structure remained stable within the temperature range relevant to most food processing and storage conditions (<200 °C).

### 3.5. Fourier-Transform Infrared Spectroscopy

FTIR spectroscopy was used to analyze the chemical structure of the microspheres and the interaction between PRE and the wall material. By comparing the FTIR spectra (Figure 2c) of the polyphenol-loaded microspheres (S-C Ms-1) and the blank microspheres (S-C Ms-0), both showed the characteristic absorption peaks of sodium alginate and chitosan, which clearly confirmed that jujube pulp bioactive compounds had been successfully embedded in the microsphere matrix composed of SA and CS. The FTIR spectrum of the non-crosslinked SA/CS physical mixture is shown in Supplementary Figure S1. It is evident from this figure that the spectrum of the blank microspheres (S-C Ms-0) showed a broad absorption peak near 3421 cm^−1^, which was attributed to the overlap of the stretching vibration absorption peak between the wall material sodium alginate and the large amount of hydroxyl (-OH) and amino (-NH_2_) groups on the chitosan molecular chain. The broad peak shape indicates the existence of strong hydrogen bonding between the molecules. The strong absorption peak near 1635 cm^−1^ is the result of the overlap between the asymmetric stretching vibration of the carboxyl radical ion (-COO^−^) of sodium alginate and the chitosan amide I band (C=O stretching). In addition, the absorption peak near 1060 cm^−1^ was the characteristic signal of the stretching vibration of C-O-C glycosidic bonds of the polysaccharide skeleton [52]. The key point is that no new characteristic absorption peaks of PRE were observed in the spectra of polyphenol-loaded microspheres (S-C Ms-1), indicating that PRE was physically embedded in the gel network rather than bound to the wall material through chemical bonds. However, compared with S-C Ms-0, the absorption peak at 3421 cm^−1^ was much wider. This is due to the large number of phenolic hydroxyl groups in the molecular structure of PRE, which strongly interact with the hydroxyl and amino groups of the wall material through hydrogen bonding, which is an important basis for stable embedding. In the “fingerprint region” from 1700 cm^−1^ to 900 cm^−1^, the spectral profile of S-C Ms-1 became more complex, with new shoulder peaks and changes in absorption intensity. These changes could be attributed to the reconstruction of intermolecular interactions, resulting in the superposition of characteristic absorption and wall spectra such as benzene ring skeleton vibrations and phenolic hydroxyl C-O stretching vibrations in the polyphenol. These subtle but definite changes confirmed the successful introduction of jujube pulp bioactive compounds into the microspheres. After drug loading, the characteristic peak located at 1635 cm^−1^ also shifted and changed its intensity. This indicated that the introduction of PRE interfered with the original intermolecular forces of the wall material, and the equilibrium in the system was reconstructed through electrostatic attraction or hydrogen bonding (e.g., between the phenolic hydroxyl group of PRE and the carboxyl group of SA), which further confirmed the successful recombination of PRE and the wall matrix [49,53].

### 3.6. X-Ray Diffractometer Analysis

XRD analysis was used to determine the crystal structure state of the raw material and the prepared microspheres. The results showed that the XRD pattern of PRE showed a typical diffuse steam peak at 2θ = 23.2°, indicating that it was amorphous. The SA/CS physical mixture also shows a broad amorphous peak at 2θ = 20.08°. Specifically, the raw material PRE showed a typical amorphous dispersion peak in the XRD pattern, with the main peak located at 2θ = 20° to 25°, but there was a weak broad peak at about 22.2°, suggesting that it contained a small amount of crystalline components or local ordered structures (Figure 2d). The sodium alginate/chitosan (SA/CS) wall mixture showed typical semi-crystalline characteristics of polysaccharides, with two broad diffraction peaks at 2θ ≈ 10.9° and 22.2°, of which the peak at 22.2° had a higher intensity [54]. The XRD patterns of the blank (S-C Ms-0) and drug-loaded (S-C Ms-1) microspheres prepared using the ion-gel method only showed broad amorphous dispersion peaks, and the intensity decreased significantly, indicating that the original semi-crystalline structure of the wall material was destroyed during pelleting [55]. In particular, the weak crystallization signal of the PRE raw material was not detected in the drug-loaded microspheres, indicating that PRE had been completely transformed into an amorphous state during the embedding process, and it was uniformly dispersed in the polysaccharide matrix in the molecular form to form a stable amorphous solid dispersion system [55,56]. This amorphous transformation is of great significance to improve the bioavailability of jujube bioactive compounds, as amorphous substances can dissolve without overcoming lattice energy, thus generally exhibiting higher apparent solubility and dissolution rate [55,56,57]. Studies have shown that the transformation of natural jujube bioactive compounds such as quercetin into amorphous forms through microcapsule or solid dispersion technology can effectively enhance their oral absorption and biological activity. Therefore, the amorphous drug-loaded microspheres constructed in this study are expected to significantly improve the dissolution and absorption efficiency of jujube bioactive compounds in the gastrointestinal tract. In addition, the SA/CS polysaccharide matrix can also protect the polyphenol structure during digestion, reduce its degradation, and further improve the bioavailability [55,56,57].

### 3.7. Micromorphology of Microspheres

**Figure 3:**
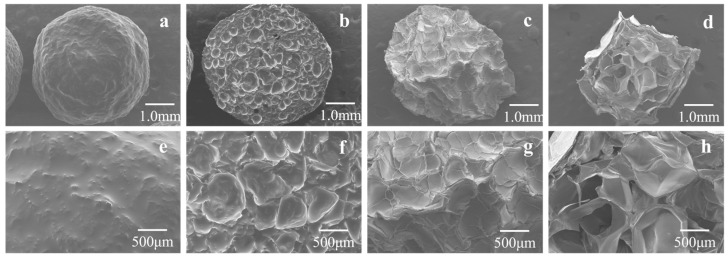
The microstructures of the two types of microspheres: (**a**,**b**) the overall morphology and cross-sectional structure of S-C MS-1; (**c**,**d**) the overall morphology and cross-sectional structure of SA Ms; (**e**,**f**) the SEM images of the overall and cross-sectional views of S-C MS-1 at different magnifications; and (**g**,**h**) the SEM images of the overall and cross-sectional views of SA Ms at different magnifications.

The microstructure of microspheres is the physical basis that determines their mechanical strength, embedding efficiency, and release kinetics [58]. Figure 3 shows the distinct morphology of single alginate microspheres (SA Ms) and composite microspheres (S-C Ms) after freeze-drying. SA Ms (Figure 3d,g,h) showed obvious irregular flattening and surface collapse, and their cross-sections did not form a continuous 3D network, but showed a disordered lamellar stacking structure with huge gaps between the layers. This structural collapse indicated that the single Ca^2+^–alginate coordination network was not mechanically strong enough to resist the capillary stress caused by water sublimation during freeze-drying, leading to the structural retraction of the gel skeleton [45]. This loose and open lamellar structure cannot effectively block the invasion of gastric acid, which also explains the high burst release of SA Ms in the gastric phase, as observed above [45,59]. In contrast, S-C Ms (Figure 3a,b,e,f) exhibited excellent structural integrity. The overall shape of the composite microspheres was regular, and the surface was rough (Figure 3a) but compact and continuous, with no cracks, which confirmed that chitosan and sodium alginate formed a solid polyelectrolyte complex (PEC) shell at the interface [60,61]. In the internal structure (Figure 3b,e), S-C Ms presented a uniform, connected honeycomb-like porous network with thick and dense walls [45,58]. The formation of this structure was attributed to the strong electrostatic interaction between the amino group of chitosan and the carboxyl group of sodium alginate. This additional physical crosslinking significantly enhanced the rigidity of the polymer matrix, enabling it to support the 3D skeleton and withstand the structural stress during drying [61]. Local magnification (Figure 3f) further shows that the pore size distribution is uniform and the pore wall is dense. This dense honeycomb network not only provides a large amount of microencapsulation space for PRE to improve the embedding rate but also provides a tortuous diffusion path for the release of more active molecules, thus giving the microspheres excellent sustained release performance [45,59,60,61].

### 3.8. Analysis of pH-Responsive Swelling Behavior and Kinetic Mechanism

**Figure 4:**
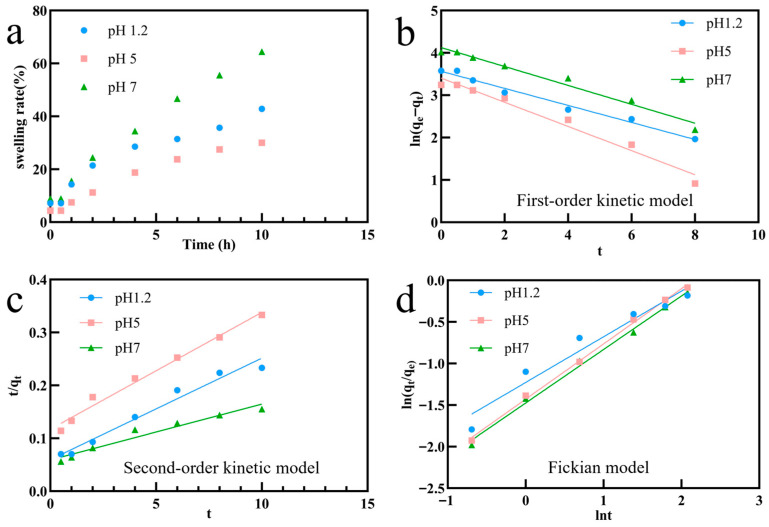
Swelling behavior and kinetic modeling of S-C MS-1 at different pH levels (1.2, 5, and 7). (**a**) Swelling rate over time; (**b**) First-order kinetic model fitting; (**c**) Second-order kinetic model fitting; (**d**) Fickian diffusion model fitting.

The swelling behavior of microspheres is a key indicator to evaluate their structural stability and predict their drug release characteristics [62]. In this study, the swelling kinetics of S-C Ms under different pH conditions was measured using the gravimetric method. Figure 4a shows that the microspheres have significant pH-dependent and time-dependent swelling characteristics, and the equilibrium swelling rate at 10 h shows a non-monotonic trend: 64.44%, 42.86%, and 30.00% at pH 7.0, pH 1.2, and pH 5.0, respectively. This specific “U-shaped” swelling difference reveals a complex molecular interaction mechanism between sodium alginate (SA) and chitosan (CS): At pH 5.0, based on the dissociation constants of SA and CS, the carboxyl group of SA and the amino group of CS are both highly ionized, forming the strongest electrostatic attraction to construct the most compact three-dimensional network, thereby limiting the penetration of water molecules to the maximum [63,64]. However, at pH 1.2, which is close to the pH of gastric juice, although the carboxyl group of SA was protonated to form an insoluble alginate skeleton, the loss of negative charge weakened the electrostatic crosslinking interaction between SA and CS, resulting in a slightly loosened gel network compared with that at pH 5.0, resulting in a moderate swelling rate [63]. When the environment changed to pH 7.0, which was close to the pH of intestinal fluid, CS deprotonation occurred, leading to structural disintegration of the complex. At the same time, the electrostatic repulsion between SA chain segments increased sharply, which promoted the expansion of the polymer network and water absorption, providing a structural basis for the intestinal release of polyphenols [63,64,65].

To further analyze the mechanism of swelling, quasi-first-order kinetic models, quasi-second-order kinetic models, Schott’s pseudo-second-order kinetic model, and the Korsmeyer–Peppas model were fitted to the data (Figure 4b–d and Table 2) [66,67]. The Korsmeyer–Peppas model (Mt/M∞ = kt^n^) is particularly useful for analyzing polymeric release systems, where the diffusional exponent (*n*) characterizes the transport mechanism. This model encompasses the Higuchi model (Fickian diffusion) when *n* = 0.5 and the Hixson–Crowell characteristics (erosion-controlled) in specific limit cases.

The results showed that the swelling mechanism was regulated by environmental pH: At pH 1.2, R^2^ of the proposed first-order kinetic model was 0.9830, which was better than that of the Fickian model (R^2^ = 0.9504), suggesting that the polymer chain segments were stiff in the strongly acidic environment, and the swelling process was mainly controlled by the diffusion rate of solvent to the gel interface. However, under pH 5.0 and pH 7.0 conditions, the Fickian model showed the best goodness of fit, with R^2^ as high as 0.9976 and 0.9950, respectively, and the model parameter *n* values were 0.6639 and 0.6434, respectively. These values fall within the range of 0.5 < *n* < 0.85, indicating anomalous (non-Fickian) transport.

These results confirmed that the swelling of the microspheres in weak acidic and neutral environments follows a non-Fickian diffusion mechanism (anomalous transport), in which the chain relaxation rate is comparable to the solvent diffusion rate. The deviation from the ideal Higuchi behavior (*n* = 0.5) highlights that the rearrangement of the SA/CS chains plays a significant role comparable to water diffusion [67]. Furthermore, while the Hixson–Crowell model typically describes erosion-controlled release, the “U-shaped” swelling trend and the anomalous transport mechanism indicate that, at pH 7.0, the microspheres undergo significant swelling coupled with partial matrix disintegration. This behavior aligns with the “moderate gastric contraction and high intestinal swelling” strategy, providing a kinetic rationale for the sustained release profiles observed in the simulated digestion studies [65,66,67].

### 3.9. Antimicrobial Activity

**Figure 5:**
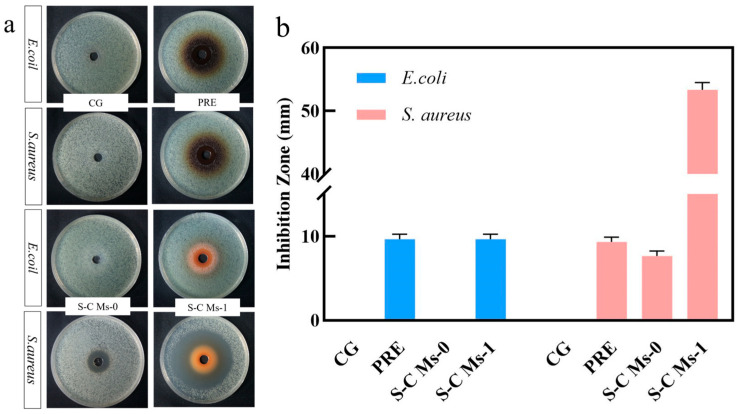
Inhibition zone images (**a**) and range values (**b**) of blank S-C Ms and drug-loaded microspheres against *E. coli* and *S. aureus*.

The SA-CS microsphere-loaded jujube pulp polyphenol system showed significant antibacterial activity against foodborne pathogens (Figure 5) [68,69]. Figure 5a shows the morphology of inhibition zone visually, and Figure 5b quantifies and compares the antibacterial efficacy of each group. As shown in Figure 5, the free polyphenol group (PRE) exhibited a distinct inhibition zone, confirming the intrinsic antibacterial potential of the jujube pulp bioactive compounds. However, a significant darkening (dark brown color) was observed in the inhibition zone of the PRE group. This phenomenon is attributed to the rapid oxidation of free polyphenols when directly exposed to the oxygen-rich culture environment, which may compromise their long-term stability and bioactivity [70]. The results showed that the inhibition zone diameters of polyphenol-loaded microspheres (S-C Ms-1) against *Escherichia coli* (*E. coli*) and *Staphylococcus aureus* (*S. aureus*) were significantly larger than those of blank microspheres (S-C Ms-0) and phosphate buffer control group (CG) [71].

This enhanced antibacterial activity was due to the synergistic mechanism of polyphenols, chitosan, and the sustained-release structure of microspheres. First, S-C Ms-0 (blank microspheres) showed some basal antibacterial activity, which confirmed that chitosan (CS), as a positively charged polyelectrolyte, can be adsorbed on the negatively charged bacterial cell wall (and interfere with cell membrane permeability) through electrostatic interactions [72]. However, the inhibition zone of S-C MS-1 was significantly larger than that of the control group, suggesting that jujube bioactive compounds contributed predominantly to the antibacterial activity of the microspheres. The abundant phenolic hydroxyl groups in jujube bioactive compounds can potentially disrupt bacterial cell membrane integrity by increasing membrane permeability and may further interfere with intracellular energy metabolism [68].

More importantly, the microsphere structure enables the sustained-release delivery of PRE. Unlike free polyphenols (CG), which are prone to rapid diffusion or oxidation, S-C Ms-1 can act as a “reservoir” of PRE to continuously release the active components in the medium, thereby maintaining local concentrations above the minimum inhibitory concentration (MIC) around the bacteria for a long time, significantly prolonging the action time [73]. In addition, it was observed that the inhibitory effect of microspheres on *S. aureus* (Gram-positive bacteria) was slightly better than that on *E. coli* (Gram-negative bacteria), which may be related to the blocking effect of the outer membrane of Gram-negative bacteria on polyphenol penetration.

In conclusion, S-C Ms-1 not only retained the biological activity of PRE but also achieved better antibacterial performance than free polyphenols through the carrier-assisted effect, demonstrating its potential application as a natural food preservative [71,72,73].

### 3.10. In Vitro Digestion and Release Behavior and Kinetic Mechanism Analysis

**Figure 6:**
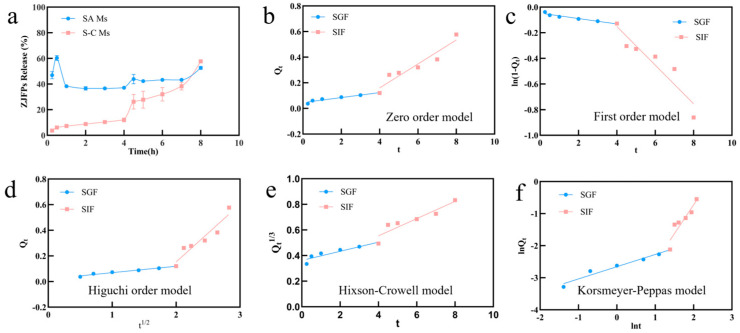
The in vitro digestion simulated release curves (**a**) and kinetic models (**b**–**f**) of different wall material pellets: (**b**) zero-order model; (**c**) first-order model; (**d**) Higuchi model; (**e**) Hixson–Crowell model; and (**f**) Korsmeyer–Peppas model.

To investigate the stability and release characteristics of polyphenol-loaded sodium alginate–chitosan composite microspheres (S-C Ms) in the digestive system, a static in vitro simulated digestion model was used to monitor the changes in the release concentration of PRE in simulated gastric juice (SGF, pH 1.2) and simulated intestinal juice (SIF, pH 6.8) (Figure 6a). A variety of kinetic models were used to elucidate the release mechanism (Figure 6b–f and Table 2) [74]. From the release curve (Figure 6a), it is evident that the single alginate microspheres (SA Ms) and the composite microspheres (S-C Ms) exhibit quite different release behavior. SA Ms showed a “burst release effect” within the first 30 min after the SGF injection, with the release rate of PRE rapidly rising to 62%, and then maintaining at about 41%, although it decreased slightly. This indicated that, although the single SA network contracted in the strong acid environment, due to the lack of a dense external barrier and high porosity, it could not effectively retain surface adsorption and shallow polyphenols, leading to the premature loss of active ingredients at the gastric stage [33,65].

In contrast, S-C Ms exhibited excellent gastric protection and intestinal sustained-release properties. During the SGF phase (0–4 h), the polyphenol release rate of S-C Ms was always maintained at a very low level (<12%). This was attributed to the formation of a compact polyelectrolyte complex (PEC) membrane between chitosan and alginate on the surface of microspheres. At pH 1.2, the amino protonation of chitosan (-NH_3_^+^) and the unreacted carboxyl group of sodium alginate form a stronger electrostatic barrier, and the gel network within SA is protonated and contracted, which limits the diffusion of polyphenols. When the microspheres were transferred to the SIF stage (4–8 h), the polyphenol release rate of S-C Ms showed a steady upward trend and finally reached 58%, which was significantly higher than the final retention of SA Ms, achieving the targeted delivery goal of “gastric protection–intestinal release” [74].

To further analyze the release mechanism, zero-order, first-order, Higuchi, Hixson–Crowell, and Korsmeyer–Peppas models were fitted to the release data (Table 3) [75]. At the SGF stage, the release kinetics of S-C Ms showed the best fit to the Higuchi model (R^2^ = 0.9747), indicating that the trace release at this stage was mainly controlled by the Fickian diffusion mechanism. This was further confirmed by the fitting results of the Korsmeyer–Peppas model, with a diffusion index of *n* = 0.3815 (*n* < 0.45), indicating that the release of PRE in the strongly acidic dense network was only due to the physical diffusion driven by the concentration gradient rather than the dissolution of the skeleton. Entering the SIF stage, the release mechanism undergoes a fundamental transformation. Kinetic parameters showed that both the zero-order kinetic model (R^2^ = 0.9036) and Higuchi model (R^2^ = 0.8966) showed good fit, which meant that S-C Ms could release PRE at a relatively constant rate in the intestine and had good sustained release characteristics. Noteworthily, the diffusion index of the Korsmeyer–Peppas model skyrocketed to *n* = 1.7930 (*n* > 0.89), indicating that this stage belongs to the Super Case II transport mechanism. Its mechanism is as follows: at an environment pH of 6.8, the protonation of SA carboxyls causes electrostatic repulsion, and CS causes the protonation of polyelectrolyte complex (PEC) film solution. The relaxation of the polymer chain and the erosion of the polymer skeleton become the key factors that dominate the release of PECs [61,75].

In summary, S-C Ms, through pH-responsive structural changes, effectively protect PRE in the stomach via the Fickian diffusion mechanism and enable sustained release in the intestine by the Super Case II transport mechanism. This biphasic release mechanism not only reduces the destruction of PRE activity by the acidic environment, but also maintains a high local concentration through a continuous release in the intestine, which provides a reliable carrier basis for improving the bioavailability of bioactive compounds in jujube pulp [60,61,66].

### 3.11. Discussion

In this study, SA-CS composite microspheres were successfully constructed, and the relationships between structural parameters, kinetic behaviors, and functional properties were elucidated. The FTIR analysis confirmed the strong electrostatic interactions between the carboxyl groups of SA and the amino groups of CS. This molecular-level crosslinking directly governed the macroscopic swelling kinetics. As indicated by the kinetic analysis (Section 3.8), the stiff polymer network formed at an acidic pH resulted in a Fickian-like diffusion restriction, which effectively minimized the swelling ratio. This “restricted swelling” behavior is the mechanistic cause of the “gastric protection” observed in the in vitro digestion. Although the XRD results showed that PRE was converted into a highly soluble amorphous state—which typically leads to rapid dissolution—the dense SA-CS network overcame this thermodynamic tendency. Thus, the release profile was dominated by the carrier’s kinetic control rather than the drug’s solubility, preventing burst release in the gastric phase. Furthermore, the release kinetics (anomalous transport mechanism, *n* ≈ 0.65) identified in the swelling study directly correlates with the functional bioactivity. As observed in the antibacterial assays, the microspheres exhibited a sustained inhibition zone without oxidative darkening being seen in the free polyphenol group. This suggests that the kinetic barrier not only regulates the release rate but also shields the encapsulated PRE from rapid environmental oxidation, thereby preserving its antibacterial efficacy.

Therefore, the superior performance of the S-C M system is not an isolated phenomenon but a result of an synergistic interplay: the electrostatic network dictates the swelling kinetics, which in turn regulates the release mechanism, ultimately ensuring the stability and bioactivity of PRE. Several limitations should, however, be acknowledged. While the study results demonstrate the effective encapsulation and sustained bioactivity of PRE, a more comprehensive chemical profiling of the extract is still warranted. Further characterization using advanced analytical techniques, such as HPLC or LC-MS/MS, is necessary to confirm the specific types and concentrations of individual phenolic compounds present in the *Z. jujuba* pulp. Such detailed identification would help clarify which specific molecules, or synergistic combinations of other bioactive constituents within the pulp, are primarily responsible for the observed antibacterial and functional outcomes. Addressing this would provide a deeper understanding of the molecular mechanisms underlying the bioactivity of these composite microspheres. The conclusions are based on a static in vitro digestion model, which cannot fully reproduce the dynamic physiological conditions in vivo, such as gastrointestinal motility, spatial–temporal variations in enzyme concentrations, and transit times. In addition, the long-term storage stability and performance of the composite microspheres under realistic processing and storage conditions were not systematically evaluated. Future work should therefore include in vivo metabolism and pharmacokinetic studies in animal models to verify the magnitude of bioavailability enhancement, as well as accelerated and real-time stability tests to define shelf-life characteristics and optimize storage strategies.

At the application level, this study provides a viable technological route for the high-value utilization of *Z. jujuba* fruit pulp by-products. The SA-CS composite microspheres developed here could be incorporated as functional ingredients into food matrices such as dairy products and baked goods to produce intestine-targeted, nutritionally fortified foods or dietary supplements. Such applications would not only extend the value chain of *Z. jujuba* resources, but also align with the broader agenda of the sustainable utilization of agricultural by-products, highlighting the considerable industrial relevance and commercialization potential of this encapsulation strategy.

## 4. Conclusions

This study successfully achieved the primary objective of constructing a sodium alginate–chitosan (SA-CS) microsphere system to enhance the stability and controlled release of *Z. jujuba* fruit pulp bioactive compounds. The experimental findings validated the central hypotheses regarding structural encapsulation and pH-responsive mechanisms: First, the effective electrostatic complexation between SA and CS was confirmed to convert the crystalline PRE into an amorphous state, verifying the hypothesis that the carrier matrix can modulate the physical state of the drug to potentially improve bioavailability. Second, this study validated the pH-responsive release hypothesis. The microspheres demonstrated a distinct “gastric protection–intestinal release” behavior. This was mechanistically supported by the kinetic analysis, which revealed that the stiff polymer network restricted diffusion in acidic media (gastric protection), while the relaxation of the matrix facilitated rapid release in neutral environments (intestinal delivery). In summary, the SA-CS microsphere system fulfills the design requirements for an oral delivery system by effectively overcoming the instability of free polyphenols. This study provides a validated theoretical basis for utilizing polyelectrolyte complexes in the high-value application of food-derived functional ingredients.

## Figures and Tables

**Table 1:** The particle size distribution and polydispersity index (PDI) of different samples.

Sample ID	Volume Mean Diameter (D{4,3}, μm)	Polydispersity Index (PDI)	Distribution Characteristics Evaluation
S-C Ms-1	1390 ± 4.17	0.052 ± 0.001	Highly monodisperse
S-C Ms-0	1510 ± 4.38	0.081 ± 0.001	Monodisperse
SA Ms-0	1081 ± 2.71	0.584 ± 0.001	Polydisperse

**Table 2:** The S-C MS-1 swelling kinetic parameters at different pH.

Kinetic Model	Equation	Parameters	pH 1.2	pH 5.0	pH 7.0
Pseudo-first-order	$Q_{t}=Q_{e}(1-e^{{-k}_{1}t}$)	$k_{1}(h^{-1})$	0.2012 ± 0.0002 ^b^	0.2855 ± 0.004 ^a^	0.2232 ± 0.0006 ^ab^
		$R^{2}$	0.983	0.9735	0.9742
Pseudo-second-orde	$\frac{t}{Qt}=\frac{1}{K_{2}Q_{e}^{2}}+\frac{t}{Q_{e}}$	$k_{2}(g^{-1}\cdot h^{-1})$	0.0091 ± 0.0002 ^a^	0.0095 ± 0.0003 ^a^	0.0041 ± 0.0003 ^b^
		$R^{2}$	0.9727	0.9848	0.9521
Korsmeyer–Peppas	$\frac{M_{t}}{M_{\infty }}=Kt^{n}$	*n*	0.5493 ± 0.0002 ^a^	0.6639 ± 0.0003 ^b^	0.6434 ± 0.0002 ^a^
		$k_{3}(h^{-n})$	0.2929 ± 0.0002 ^a^	0.2395 ± 0.0004 ^b^	0.2288 ± 0.0001 ^b^
		$R^{2}$	0.9504	0.9976	0.9950

Note: Data are expressed as mean ± SD (*n* = 3). Different lowercase letters in the same column indicate significant differences (*p* < 0.05).

**Table 3:** The S-C MS-1 in vitro simulated digestion kinetic parameters.

Dynamic Model	Parameter	Digestion	
		SGF	SIF
Zero-order model	K_1_	0.0194	0.0936
	R^2^	0.9363	0.9036
First-order model	K_2_	0.0212	0.1508
	R^2^	0.9424	0.8781
Higuchi order model	K_3_	0.0500	0.4506
	R^2^	0.9747	0.8966
Hixson–Crowell model	K_4_	0.0358	0.0673
	R^2^	0.8672	0.8661
Korsmeyer–Peppas model	K_5_	0.0703	0.0134
	R^2^	0.9658	0.8493
	N	0.3815	1.7930

## Data Availability

The data presented in this study are available on request from the corresponding author due to privacy concerns.

## Supplementary Materials

The following supporting information can be downloaded at: https://www.mdpi.com/article/10.3390/foods15030594/s1, Figure S1: FTIR spectra of the uncrosslinked Sodium Alginate/Chitosan physical mixture (SA/CS) compared to the microspheres.

## Abbreviations

The following abbreviations are used in this manuscript:

PRE	Phenolic-rich extract from *Z. jujuba* pulp
SA	Sodium alginate
CS	Chitosan
S-C Ms	Sodium alginate and chitosan microspheres
SA Ms	Sodium alginate microspheres
SA/CS	Sodium alginate/chitosan
EE%	Encapsulation rate
LC%	Loading capacity
SR	Swelling ratio
SGF	Simulated gastric fluid
SIF	Simulated intestinal fluid
FTIR	Fourier-transform infrared
SEM	Scanning electron microscope
XRD	X-ray diffraction
