# Supplementary material for: Preparation, Characterization, and In Vitro Digestion Behavior of Alginate–Chitosan Microspheres Loaded with Ziziphus jujuba Pulp
Source: Foods. 2026 Feb 6;15(3):594. doi: 10.3390/foods15030594 (PMC12896531; doi:10.3390/foods15030594)
Supplement: Supplementary file 1 [file foods-15-00594-s001.zip › foods-4079973-supplementary.pdf]

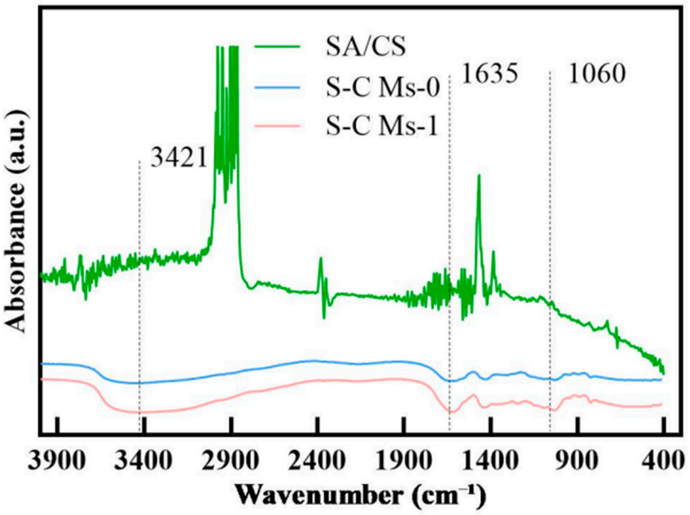

Figure S1. FTIR spectra of the uncrosslinked Sodium Alginate/Chitosan physical mixture (SA/CS) compared to the microspheres.
